# Supplementary material for: Emergence of the structure-directing role of f-orbital overlap-driven covalency
Source: Nat Commun. 2019 Feb 7;10:634. doi: 10.1038/s41467-019-08553-y (PMC6367379; doi:10.1038/s41467-019-08553-y)
Supplement: Supplementary file 1 — Supplementary Information [file 41467_2019_8553_MOESM1_ESM.pdf]

Emergence of the Structure-Directing Role of f-Orbital Overlap-Driven Covalency

Lu et al.

Supplementary Figures

Characterisation Data

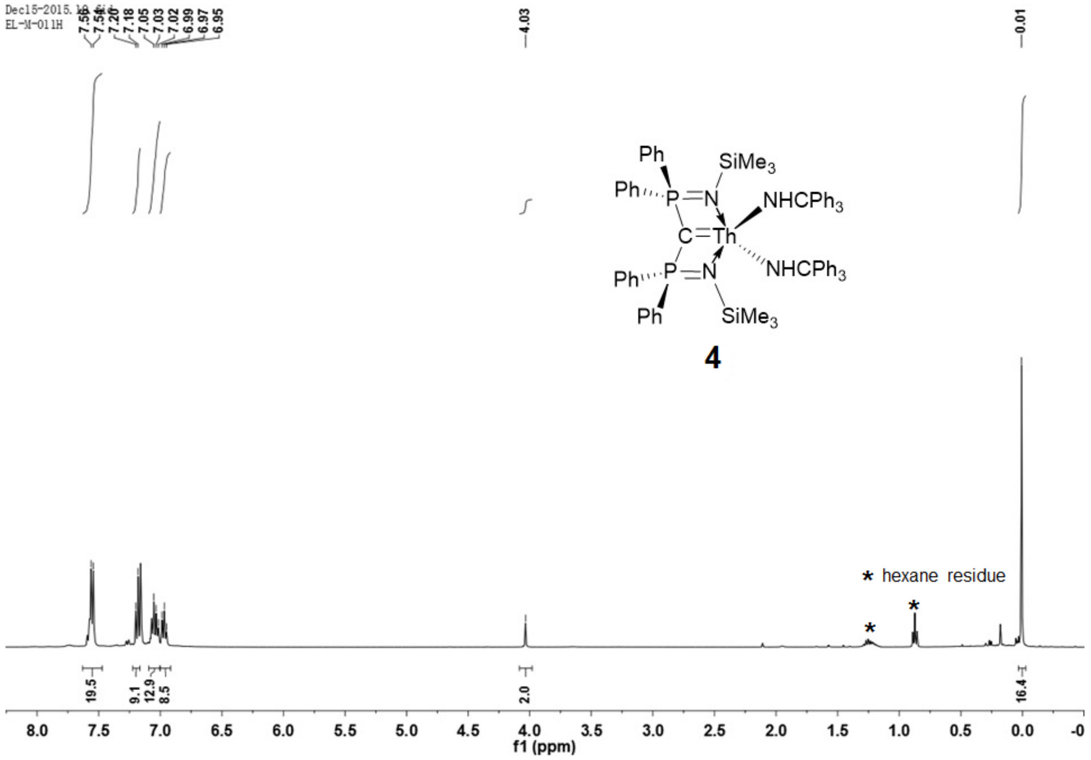

Supplementary Figure 1. ^{1}H NMR spectrum of **4** (C_6D_6, 25 °C).

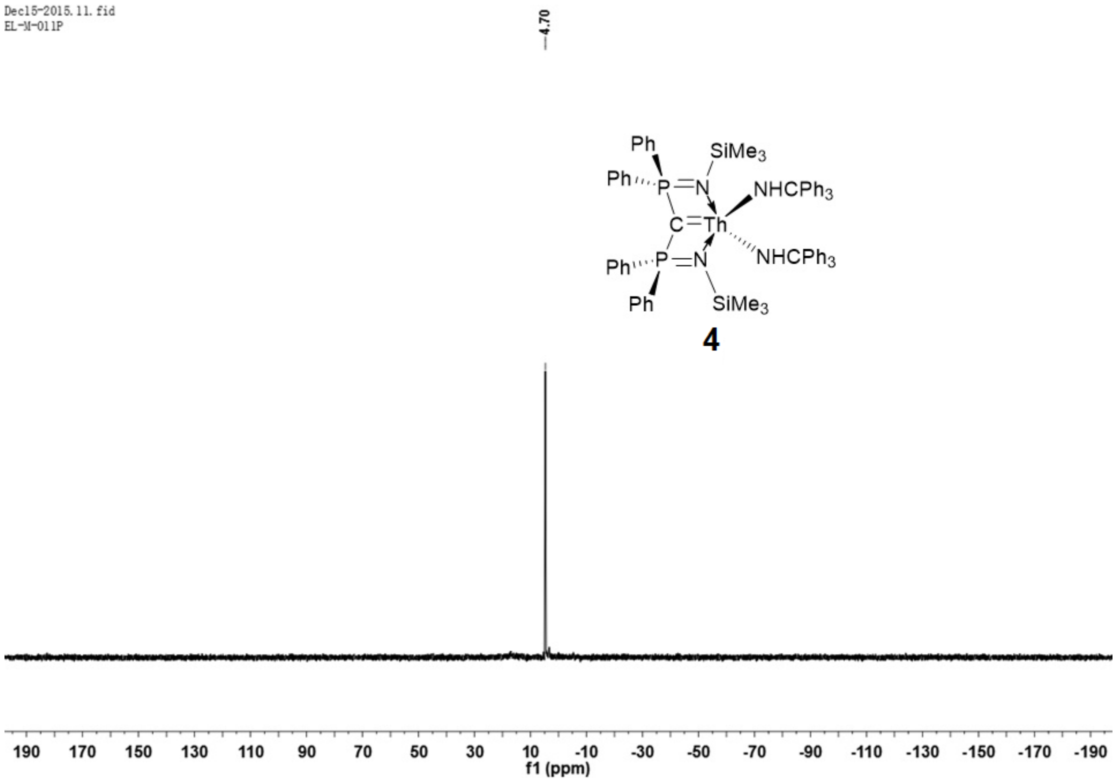

Supplementary Figure 2. ^{31}P NMR spectrum of **4** (C_6D_6, 25 °C).

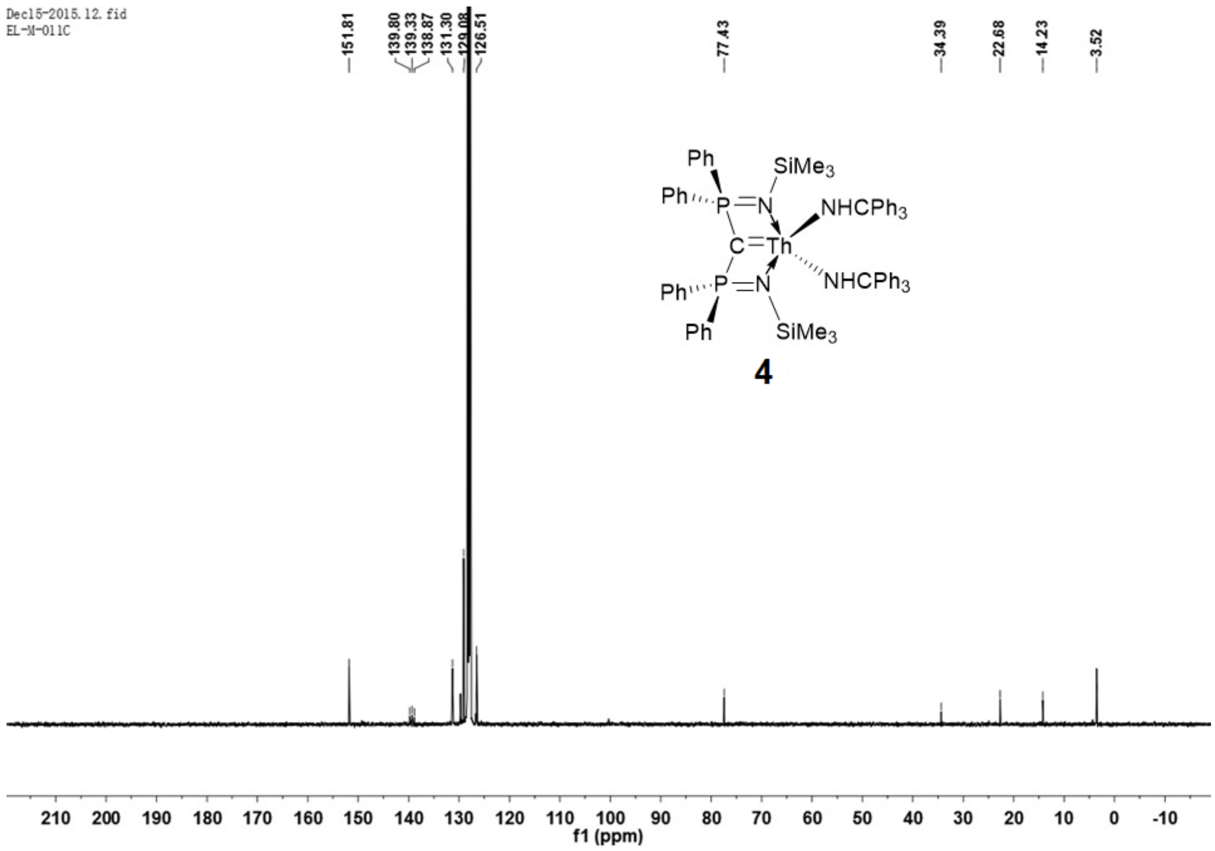

Supplementary Figure 3. ^{13}C NMR spectrum of **4** (C_6D_6, 25 °C).

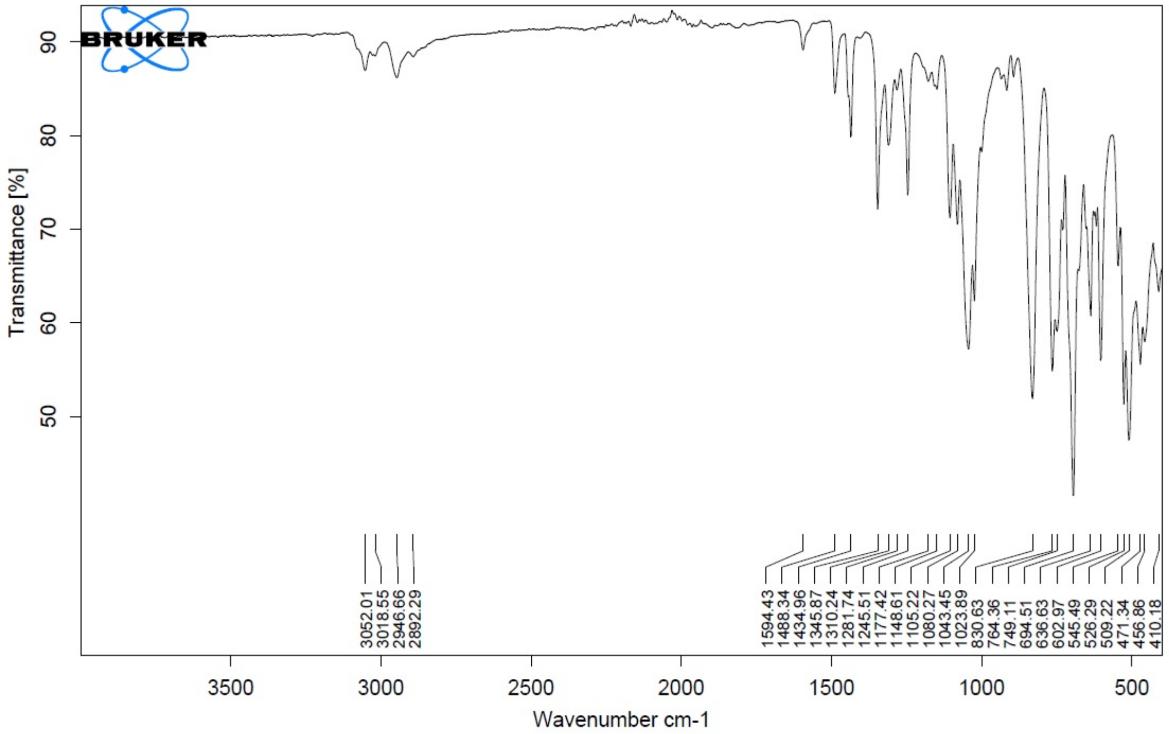

Supplementary Figure 4. FTIR spectrum of **4**.

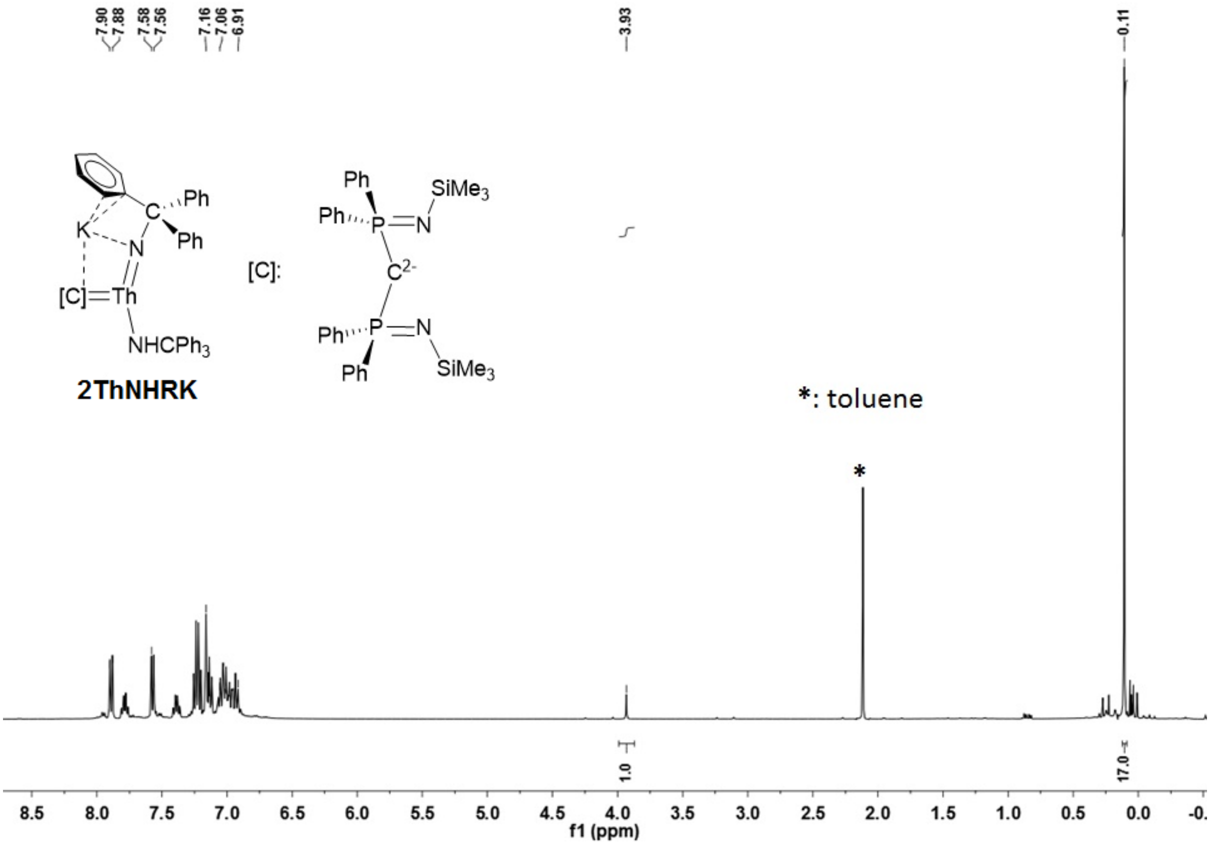

Supplementary Figure 5. ^{1}H NMR spectrum of **2ThNHRK** (C_6D_6, 25 °C).

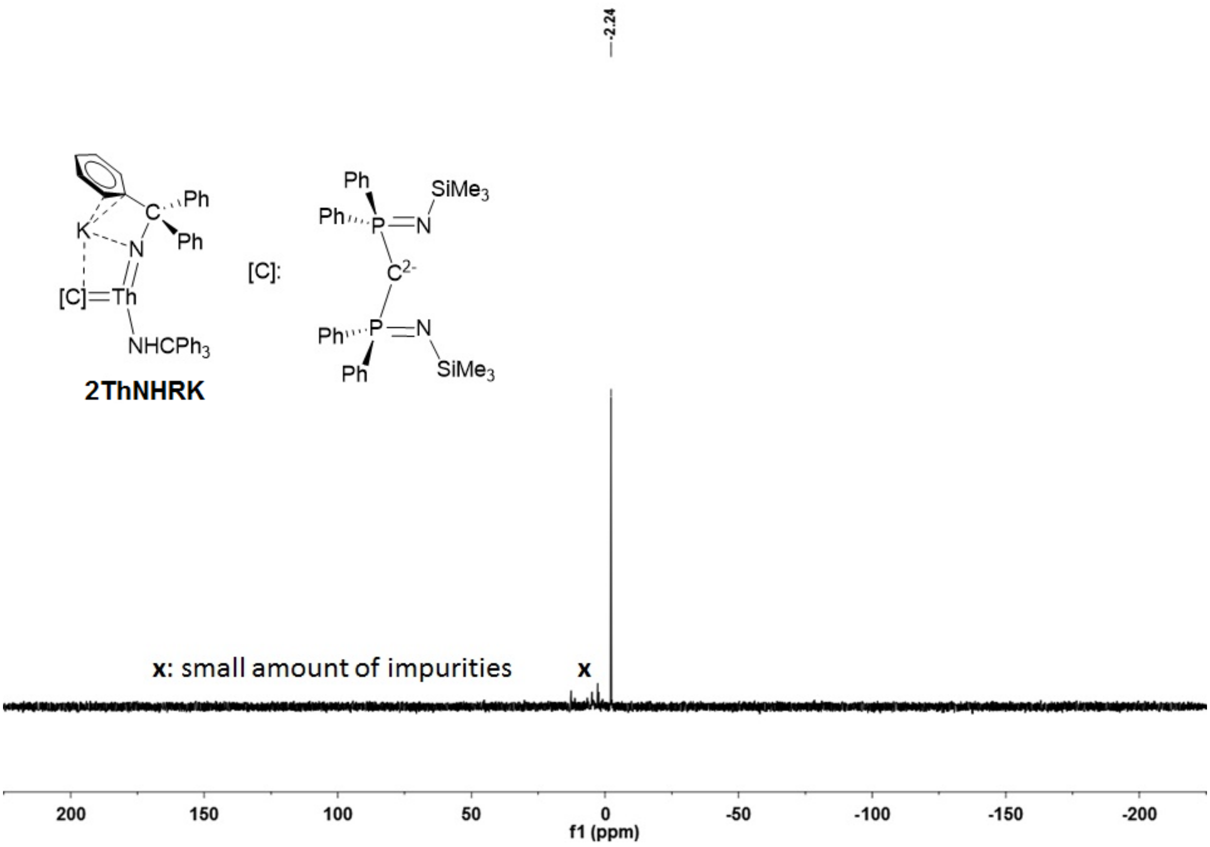

Supplementary Figure 6. ^{31}P NMR spectrum of **2ThNHRK** (C_6D_6, 25 °C).

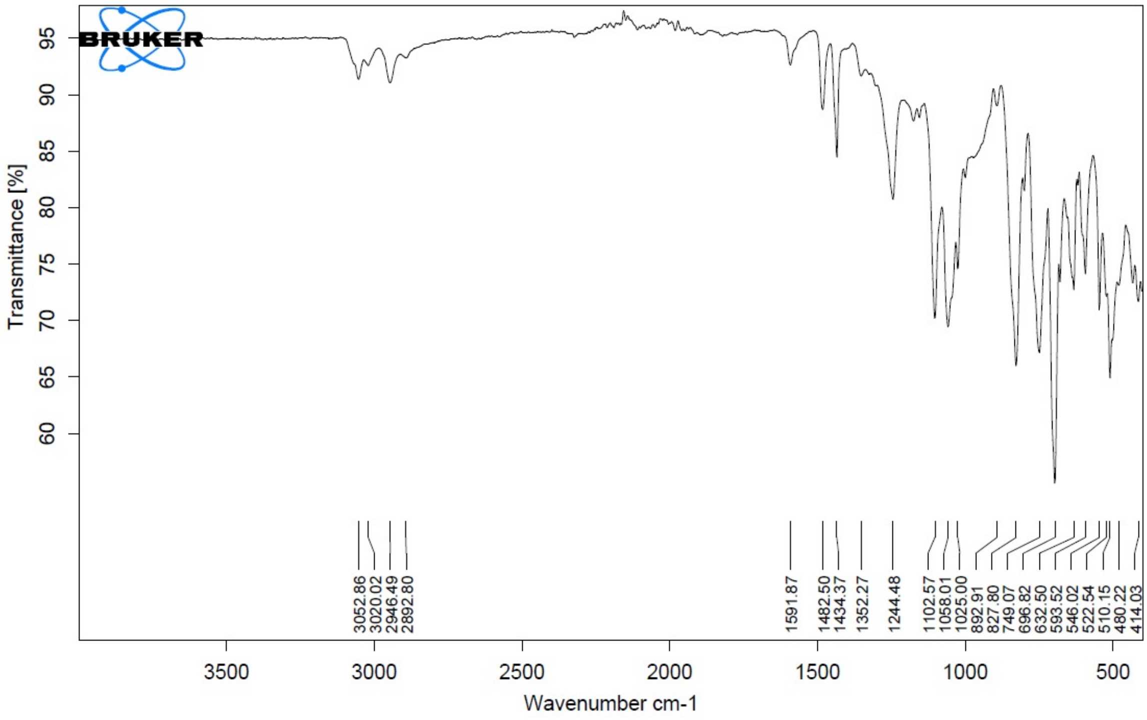

Supplementary Figure 7. FTIR spectrum of **2ThNHRK**.

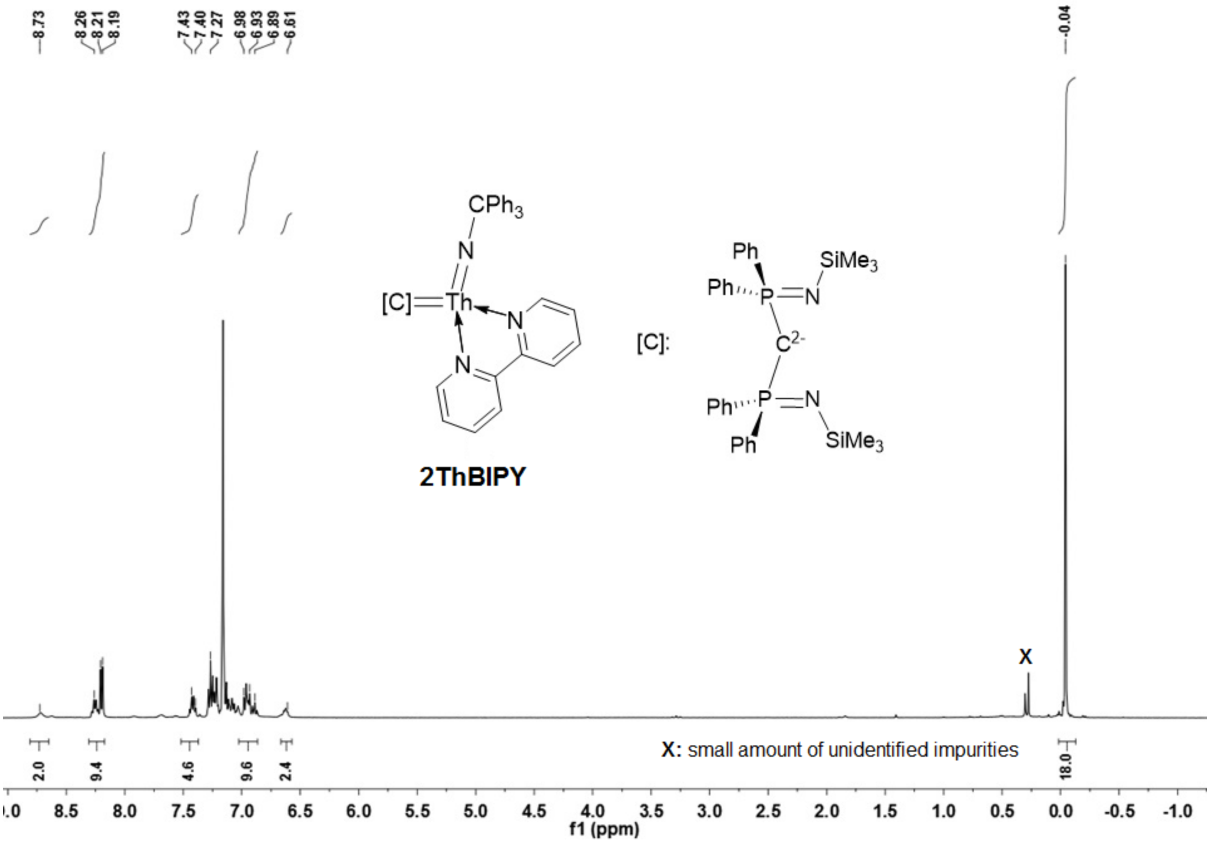

Supplementary Figure 8. ^{1}H NMR spectrum of **2ThBIPY** (C_6D_6, 25 °C).

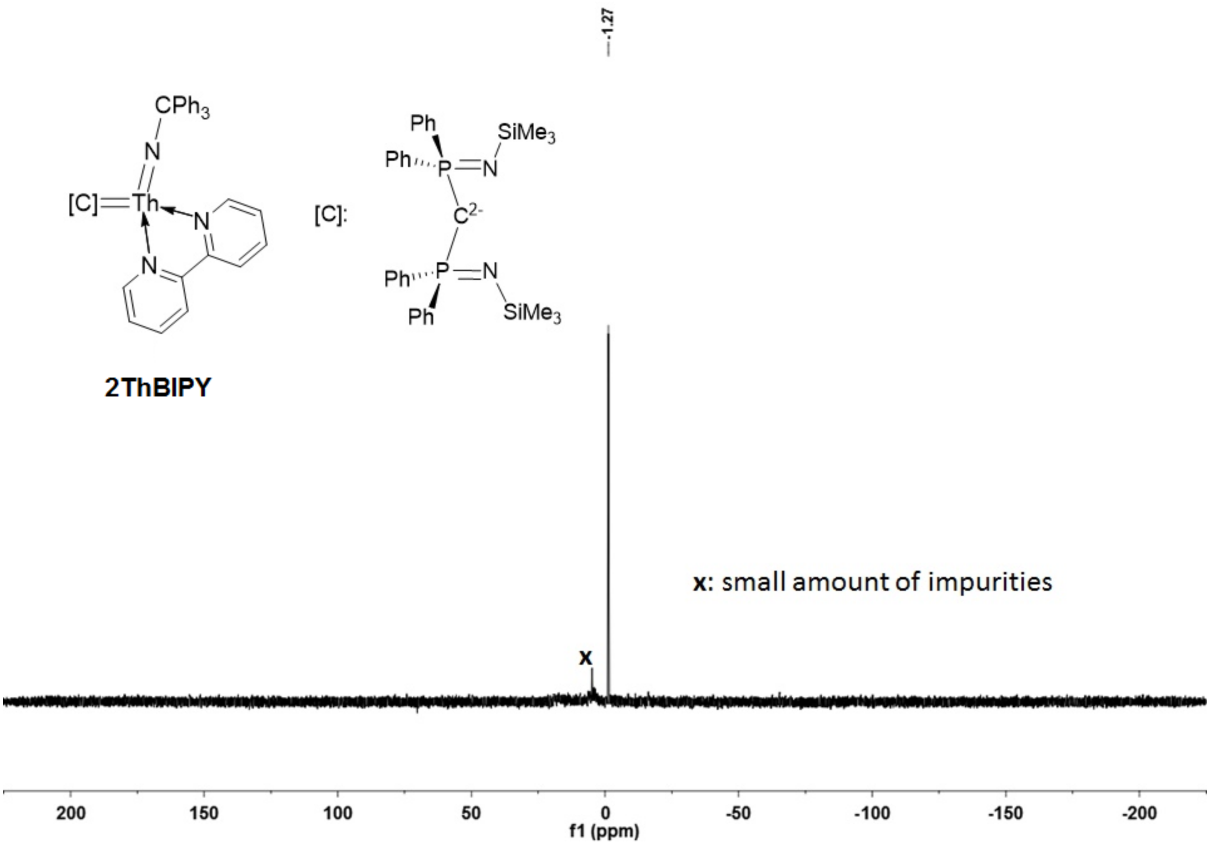

Supplementary Figure 9. ^{31}P NMR spectrum of **2ThBIPY** (C_6D_6, 25 °C).

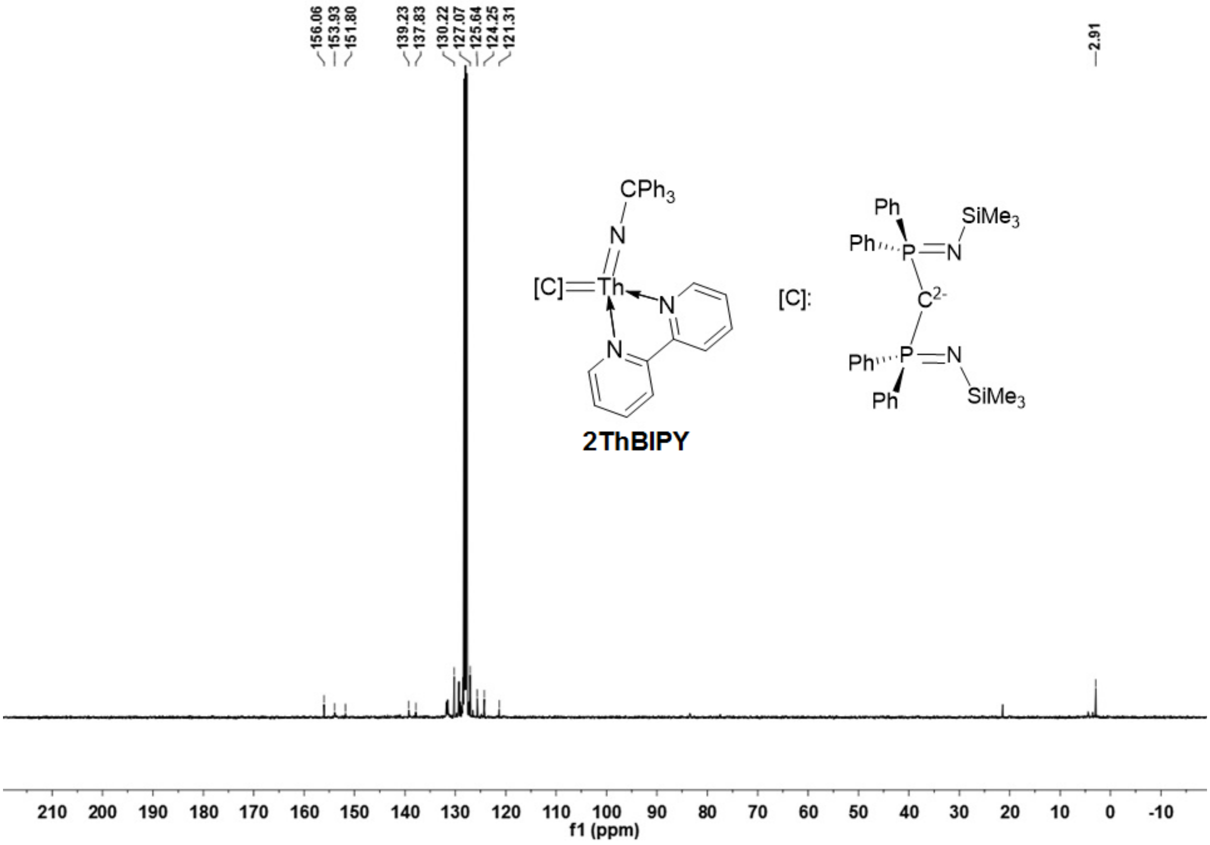

Supplementary Figure 10. ^{13}C NMR spectrum of **2ThBIPY** (C_6D_6, 25 °C).

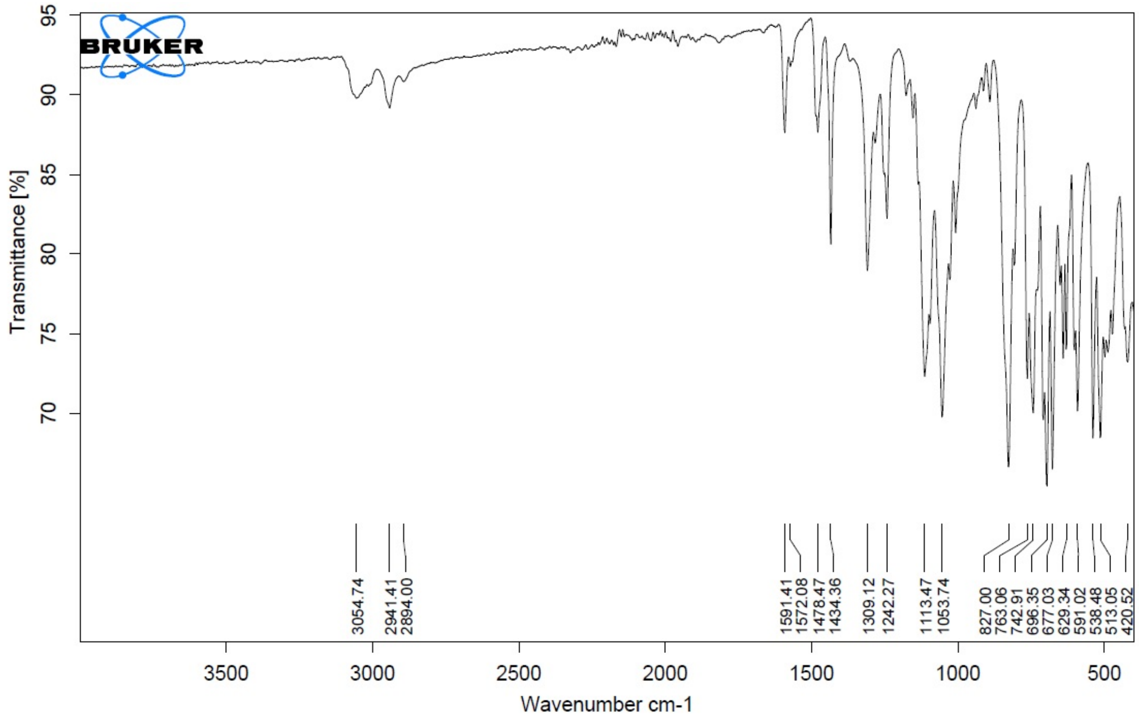

Supplementary Figure 11. FTIR spectrum of **2ThBIPY**.

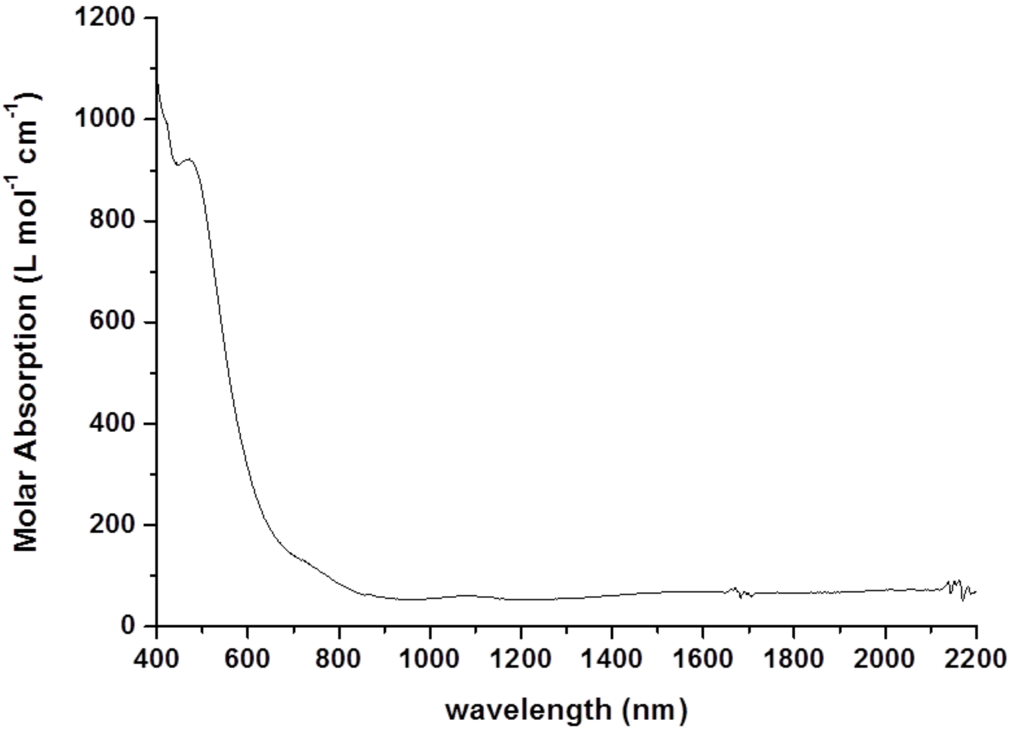

Supplementary Figure 12. Electronic absorption spectrum of **2ThBIPY**. 10 mM in toluene in 1 mm quartz cell.

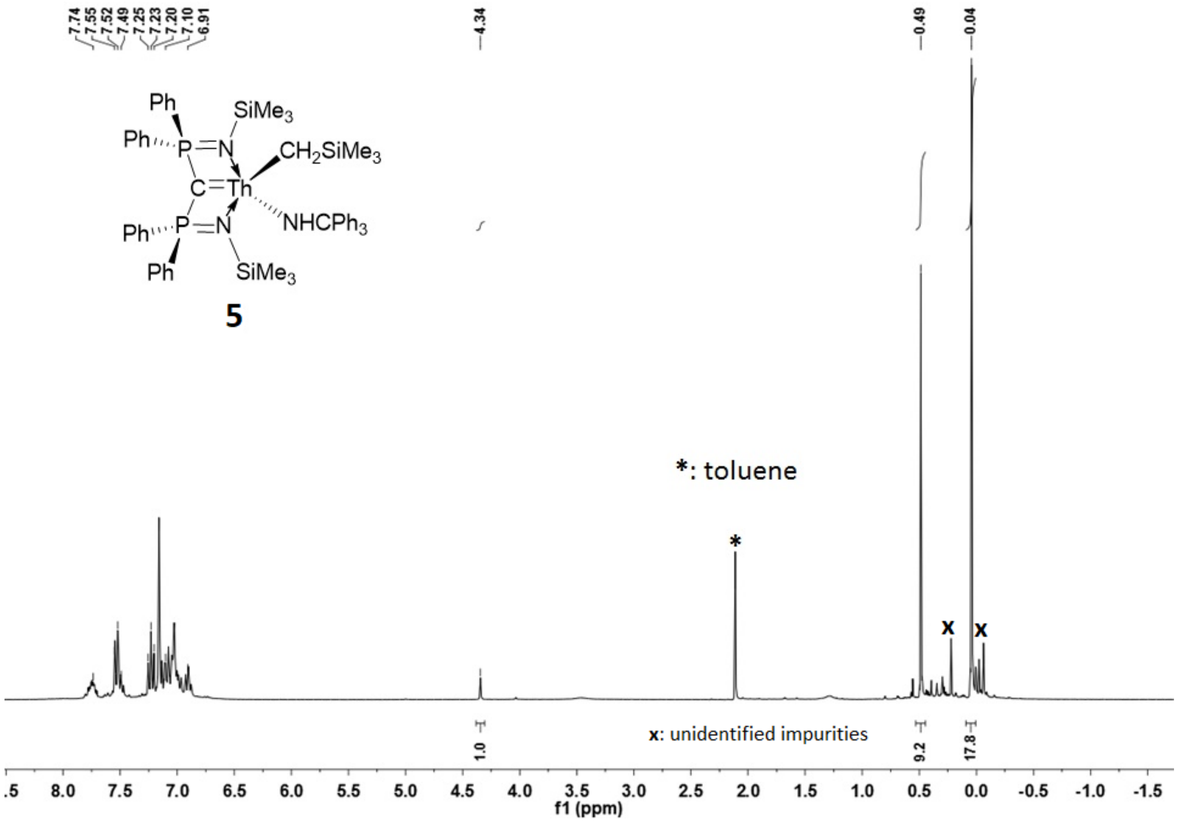

Supplementary Figure 13. ^{1}H NMR spectrum of **5** (C_6D_6, 25 °C).

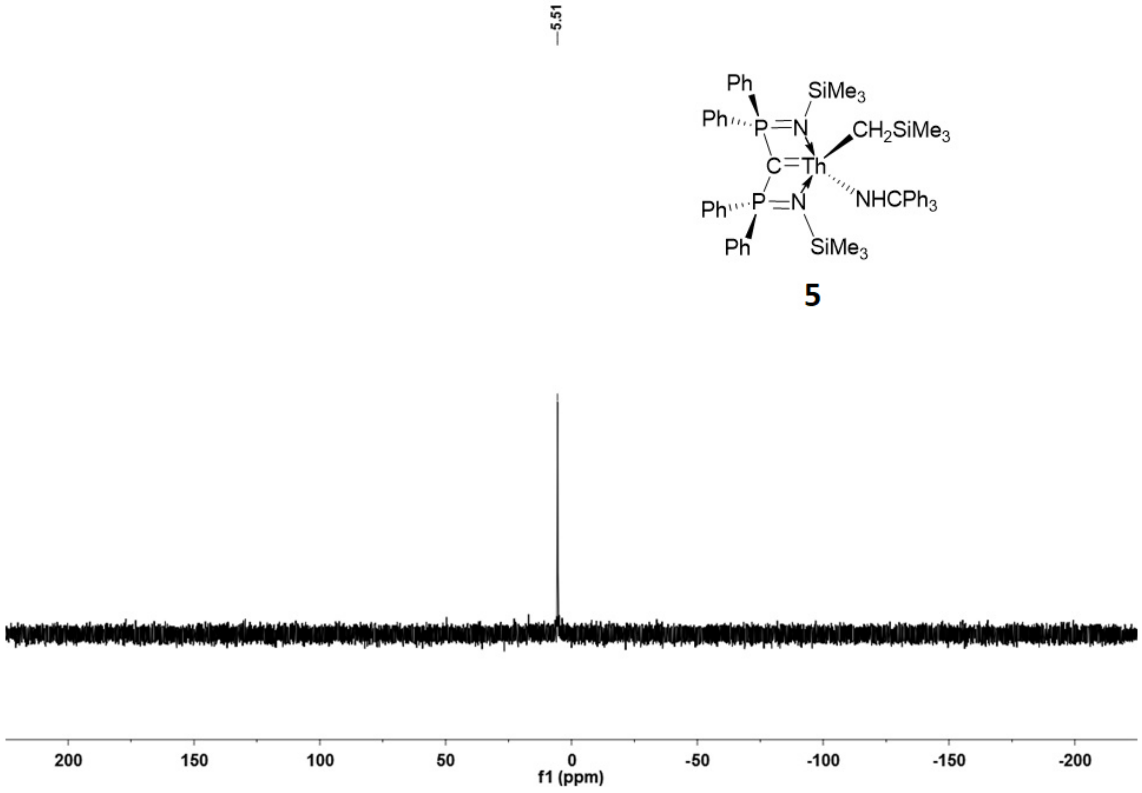

Supplementary Figure 14. ^{31}P NMR spectrum of **5** (C_6D_6, 25 °C).

Computational Figures

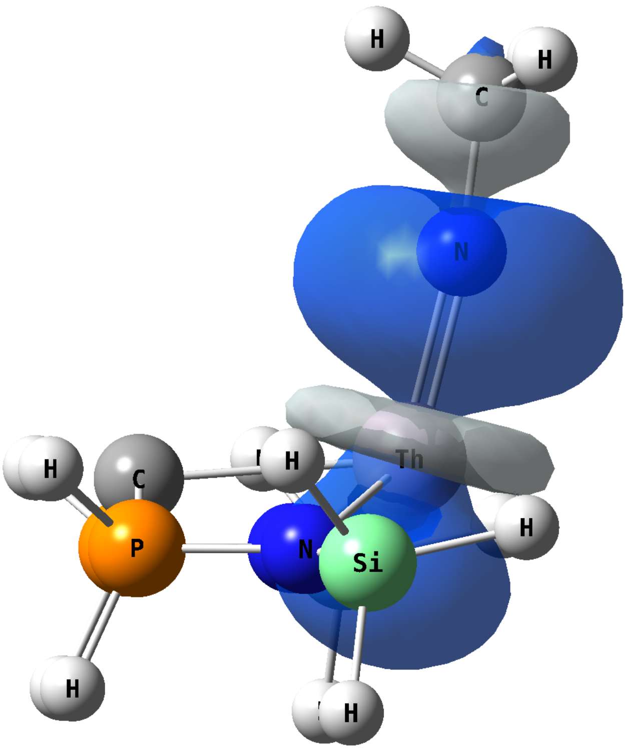

Supplementary Figure 15. NLMO for Th-N σ bond in $R_2C{=}Th^{IV}{=}NCH_3$.

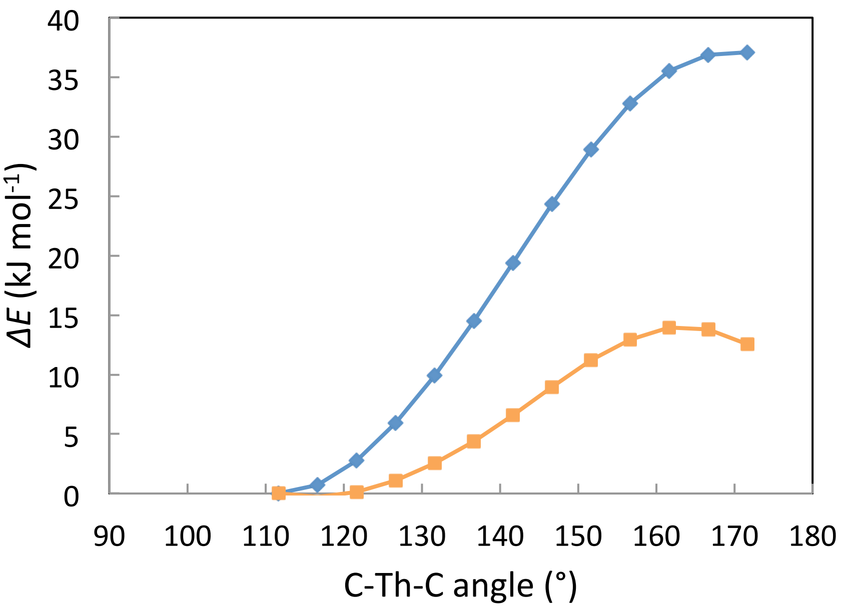

Supplementary Figure 16. SCF energy surface scan (PBE0) of the $C{=}Th^{IV}{=}C$ angle in the optimised $R_2C{=}Th^{IV}{=}C(CH_3)_2$ system (blue) and when r(Th–$C_{[C]}$) ([C] = $C(PH_2NSiH_3)_2$) is increased by 1.0 Å (orange). Energies (kJ mol^{-1}) are presented relative to the fully optimized $R_2C{=}Th^{IV}{=}C(CH_3)_2$.

Supplementary Tables

Supplementary Table 1. Optimum C=M=E angles using PBE and PBE0 DFAs for the nine model complexes.

R_2C=M=E			∠ C-M-E (degree)	
	E	M	PBE0	PBE
1'-Ce	$C(CH_3)_2$	Ce	128.3	123.6
1'-Th		Th	111.6	111.4
1'-U		U	109.8	107.7
2'-Ce	NCH_3	Ce	116.7	118.6
2'-Th		Th	108.4	107.6
2'-U		U	108.2	108.5
3'-Ce	O	Ce	165.1	162.2
3'-Th		Th	116.8	116.6
3'-U		U	176.9	176.4

Supplementary Table 2. PBE0 structural, energetic, and frequency data for the true minima (Opt) and C=M=E bending transition states (TSs) in R_2C=M=E (R = PH_2NSiH_3; M = Ce, Th, U; E = $C(CH_3)_2$, NCH_3, O).

R_2C=M=E		∠ C=M=E (°)			
E	M	Opt	TS	$E_{TS\text{-}Opt}$ (kJ mol^{-1})	TS imaginary mode (cm^{-1})
$C(CH_3)_2$	Ce†	128.3	-	-	-
	Th	111.6	169.9	37.1	37.2*i*
	U	109.8	171.7	19.5	24.8*i*
NCH_3	Ce	116.7	176.1	14.4	28.3*i*
	Th	108.4	164.7	27.1	43.0*i*
	U	108.2	154.5	13.7	27.2*i*
O	Ce	165.1	-	-	-
	Th	116.8	176.8	14.7	42.7*i*
	U	176.9	142.2	3.3	40.0*i*

† No TS could be located for R_2C=Ce=$C(CH_3)_2$, and no attempt was made to locate one for R_2C=Ce=O on the basis of Figure 3c.

Supplementary Table 3. Absolute energy changes in the σ and π NLMOs between the trans and cis conformations of $R_2C{=}M^{IV}{=}E$ (R = PH_2NSiH_3; M = Ce, Th, U; E = $C(CH_3)_2$, NCH_3, O).

$R_2C{=}M{=}E$		|Δ Energy| (eV)	
E	M	σ	π
$C(CH_3)_2$	Ce	0.336	0.130
	Th	0.302	-
	U	0.504	0.068
NCH_3	Ce	0.199	0.024
	Th†	0.125	0.068
	U	0.223	0.020
O	Ce	1.313	0.080
	Th	1.307	0.104
	U	3.231	0.108

†The smallest σ:π ratio exists for $R_2C{=}Th{=}NCH_3$ (ca. 2) and further analysis reveals that the π-orbital compositions are virtually unchanged (<0.4% in d- and f-orbital composition). Significant changes are observed for the σ-orbital compositions (>8% in d- and f-orbital composition) thus providing further evidence that the σ-orbitals are most affected by changes in ∠ C=M=E.

Supplementary Table 4. PBE0 structural data for the true minima (Opt) and $C{=}M^{IV}{=}E$ bending transition states (TS) in $R_2C{=}M^{IV}{=}E$ (R = PH_2NSiH_3; M = Ce, Th, U; E = $C(CH_3)_2$, NCH_3, O).

E	M	∠ C=M=E (°)		*r*(M=E) (Å)			*r*(R_2C=M) (Å)		
		Opt	TS	Opt	TS	$\Delta_{TS\text{-}Opt}$	Opt	TS	$\Delta_{TS\text{-}Opt}$
$C(CH_3)_2$	Ce	128.3	-	2.069	-	-	2.451	-	-
	Th	111.6	169.9	2.157	2.166	+0.009	2.489	2.677	+0.188
	U	109.8	171.7	2.112	2.098	-0.014	2.418	2.530	+0.112
NMe	Ce	116.7	176.1	1.885	1.885	0.000	2.444	2.573	+0.128
	Th	108.4	164.7	1.965	1.983	+0.018	2.491	2.641	+0.150
	U	108.2	154.5	1.915	1.926	+0.011	2.429	2.509	+0.080
O	Ce	165.1	-	1.769	-	-	2.480	-	-
	Th	116.8	176.8	1.865	1.864	-0.001	2.504	2.645	+0.141
	U	176.9	142.2	1.809	1.812	+0.003	2.489	2.468	-0.021

No TS could be located for $R_2C{=}Ce^{IV}{=}C(CH_3)_2$, and no attempt was made to locate one for $R_2C{=}Ce^{IV}{=}O$, on the basis of Figure 3c.

Supplementary Table 5. Cartesian coordinates and energies for the PBE0 optimized structures (Opt) and C=M^{IV}=E bending transition states (TS) in R$_2$C=M^{IV}=E (R = PH$_2$NSiH$_3$; M = Ce, Th, U; E = CMe$_2$, NMe, O).

E	M	Opt				TS
**C(CH$_3$)$_2$**	**Ce**	C	-0.01195	-1.51614	1.20720	NA
		N	2.06550	-0.71823	-0.28271	
		N	-2.06712	-0.76882	-0.32896	
		Si	3.68126	-0.48980	-0.82428	
		Si	-3.67406	-0.49396	-0.87198	
		P	1.50998	-1.94558	0.64526	
		P	-1.50864	-1.98462	0.61240	
		H	4.68013	-0.63504	0.28002	
		H	4.07026	-1.45693	-1.89659	
		H	3.80249	0.88457	-1.39135	
		H	1.57698	-3.18912	-0.05345	
		H	2.44859	-2.19718	1.68015	
		H	-2.46841	-2.25645	1.62234	
		H	-1.53053	-3.22727	-0.08981	
		H	-4.06127	-1.38074	-2.01132	
		H	-4.68464	-0.69887	0.21135	
		H	-3.76941	0.91803	-1.34726	
		Ce	-0.02722	0.53247	-0.13989	
		C	1.33018	3.00675	0.33474	
		H	1.39878	3.83424	-0.38736	
		H	1.78671	3.35774	1.26981	
		H	2.02115	2.22044	-0.03324	
		C	-0.06445	2.49563	0.51277	
		C	-1.15806	3.48168	0.76282	
		H	-2.15279	3.02299	0.71486	
		H	-1.07728	3.93378	1.76127	
		H	-1.14611	4.31409	0.04357	
**Energy =**		-2007.5630239				

E	M	Opt				TS			
**C(CH$_3$)$_2$**	**Th**	C	-0.10834	-1.45343	1.27122	C	-0.00246	-2.10001	-1.06113
		N	2.03760	-0.91616	-0.20113	N	-1.95099	-0.76656	0.31863
		N	-2.17279	-0.72815	-0.21978	N	1.95432	-0.83313	0.34615
		Si	3.68932	-0.78300	-0.67507	Si	-3.49891	-0.47241	1.03987
		Si	-3.77754	-0.40125	-0.75434	Si	3.52170	-0.48340	0.99340
		P	1.41771	-1.99661	0.86661	P	-1.47536	-2.24318	-0.27473
		P	-1.66643	-1.86919	0.84607	P	1.44421	-2.29698	-0.24082
		Th	-0.02078	0.43295	-0.34964	Th	0.03261	0.46268	-0.28629
		H	4.61746	-0.74399	0.49574	H	-4.61273	-0.65317	0.05962
		H	4.12192	-1.91709	-1.54724	H	-3.75306	-1.40821	2.17717
		H	3.85025	0.47771	-1.45522	H	-3.53539	0.92363	1.55172
		H	1.49796	-3.32538	0.35420	H	-1.54560	-3.20084	0.77979
		H	2.31119	-2.10428	1.96465	H	-2.51256	-2.71305	-1.11834
		H	-2.58607	-1.91166	1.92640	H	2.48873	-2.80797	-1.05046
		H	-1.85158	-3.17938	0.31420	H	1.44744	-3.25011	0.82017

		H	-4.29307	-1.44103	-1.69522	H	3.69020	-1.00761	2.38063
		H	-4.74628	-0.30181	0.37835	H	4.60088	-1.08418	0.15117
		H	-3.75404	0.90204	-1.48123	H	3.69415	0.99561	1.02197
		C	1.54271	2.71387	0.42008	C	-1.35225	2.97057	-0.07961
		H	1.64494	3.69356	-0.07182	H	-1.73115	3.44554	0.83736
		H	2.15656	2.74891	1.33257	H	-1.60895	3.64029	-0.91512
		H	2.08488	2.00395	-0.24618	H	-2.01746	2.08288	-0.21191
		C	0.09514	2.33885	0.65404	C	0.11605	2.61177	-0.02833
		C	-0.64964	3.31026	1.51976	C	1.02982	3.79853	0.08957
		H	-1.69525	3.02324	1.66498	H	2.08685	3.51138	0.09553
		H	-0.20374	3.40592	2.52103	H	0.89715	4.51385	-0.73661
		H	-0.65230	4.32340	1.08919	H	0.85468	4.36757	1.01468
**Energy =**		-1940.2013663				-1940.1872212			

**E**	**M**	**Opt**				**TS**			
**$C(CH_3)_2$**	**U**	C	-0.08385	-1.31720	1.37065	C	0.39778	-1.96673	-1.04715
		N	2.00975	-0.92247	-0.21837	N	-1.70185	-1.08637	0.41210
		N	-2.11122	-0.76959	-0.24712	N	2.09019	-0.37888	0.34378
		Si	3.62549	-0.83020	-0.80385	Si	-3.21993	-1.01631	1.23690
		Si	-3.68634	-0.55192	-0.90614	Si	3.51665	0.27917	1.06026
		P	1.42332	-1.91287	0.94220	P	-0.98037	-2.43505	-0.19532
		P	-1.62238	-1.80434	0.92257	P	1.88405	-1.89787	-0.25689
		H	4.62959	-0.72215	0.29864	H	-4.30126	-1.67588	0.44147
		H	4.00259	-2.01610	-1.63159	H	-3.16272	-1.70687	2.56082
		H	3.73965	0.38177	-1.66651	H	-3.58649	0.40740	1.45872
		H	1.45016	-3.27506	0.51963	H	-0.76390	-3.38569	0.84453
		H	2.34990	-1.96960	2.01628	H	-1.92213	-3.13148	-0.99236
		H	-2.57173	-1.79437	1.97762	H	2.98129	-2.20043	-1.10138
		H	-1.74474	-3.15840	0.49245	H	2.07459	-2.85687	0.78039
		H	-4.10750	-1.69055	-1.77723	H	3.83890	-0.36740	2.36749
		H	-4.72945	-0.39015	0.15166	H	4.70913	0.11102	0.17431
		H	-3.66516	0.68224	-1.74474	H	3.29510	1.73338	1.29306
		C	1.47780	2.71818	0.37753	C	-2.00278	2.48488	-0.50707
		H	1.55468	3.66921	-0.17062	H	-2.73095	2.74040	0.27620
		H	2.07714	2.82402	1.29281	H	-2.14775	3.19960	-1.33027
		H	2.04097	1.97954	-0.23432	H	-2.35578	1.50709	-0.90510
		C	0.04442	2.31800	0.63653	C	-0.57759	2.42795	-0.02357
		C	-0.75979	3.32872	1.39196	C	-0.00495	3.69205	0.54194
		H	-1.80070	3.02032	1.52158	H	1.03048	3.56462	0.87736
		H	-0.35220	3.50617	2.39766	H	0.00006	4.50941	-0.19419
		H	-0.76895	4.30683	0.88812	H	-0.57569	4.05897	1.40727
		U	-0.01769	0.42731	-0.30179	U	-0.04006	0.42646	-0.35230
**Energy =**		-2009.4312865				-2009.4238431			

**E**	**M**	**Opt**				**TS**			
**NCH_3**	**Ce**	C	0.00119	3.63598	1.24172	C	-0.00330	3.81499	0.27212
		C	-0.00012	-1.24587	1.25260	C	0.00106	-1.95321	-0.89703
		N	2.07423	-0.61638	-0.30734	N	-1.94878	-0.47686	0.27854
		N	0.00066	2.38415	0.54503	N	-0.00166	2.44016	-0.14771
		N	-2.07456	-0.61619	-0.30708	N	1.94959	-0.47486	0.27830
		Si	3.68179	-0.36005	-0.85368	Si	-3.44411	0.01932	0.97716
		Si	-3.68196	-0.35920	-0.85351	Si	3.44436	0.02246	0.97733

		P	1.51876	-1.73791	0.74176	P	-1.46219	-2.00641	-0.07000
		P	-1.51904	-1.73784	0.74186	P	1.46452	-2.00490	-0.07027
		H	0.88568	4.23609	0.99458	H	-0.89604	4.05512	0.86138
		H	-0.88188	4.23761	0.99316	H	0.87220	4.04867	0.88916
		H	0.00019	3.48873	2.32808	H	0.01277	4.49517	-0.58785
		H	4.67199	-0.30914	0.26589	H	-4.61409	-0.59313	0.27303
		H	4.14646	-1.41644	-1.80483	H	-3.55361	-0.36087	2.41865
		H	3.72271	0.94504	-1.57756	H	-3.55530	1.50070	0.87517
		H	1.58356	-3.04594	0.17373	H	-1.47168	-2.79756	1.11811
		H	2.45545	-1.88479	1.79890	H	-2.49063	-2.64674	-0.80696
		H	-2.45568	-1.88483	1.79903	H	2.49346	-2.64413	-0.80749
		H	-1.58392	-3.04579	0.17367	H	1.47512	-2.79609	1.11781
		H	-4.14653	-1.41459	-1.80583	H	3.55507	-0.36063	2.41797
		H	-4.67242	-0.30924	0.26588	H	4.61517	-0.58644	0.27151
		H	-3.72255	0.94660	-1.57615	H	3.55289	1.50423	0.87840
		Ce	-0.00009	0.67691	-0.25625	Ce	-0.00025	0.60650	-0.57400
**Energy =**		-1984.3859785				-1984.3804973			

**E**	**M**	**Opt**				**TS**			
**NCH_3**	**Th**	C	0.00154	3.29856	1.70881	C	0.00008	4.02361	-0.01967
		C	-0.00017	-1.38974	1.21015	C	-0.00002	-1.87184	-1.16110
		N	2.11570	-0.72463	-0.25077	N	-1.95590	-0.70528	0.35766
		N	0.00092	2.21106	0.77682	N	0.00004	2.58958	-0.10953
		N	-2.11613	-0.72379	-0.25020	N	1.95587	-0.70532	0.35766
		Si	3.75065	-0.42065	-0.69247	Si	-3.50179	-0.45111	1.09068
		Si	-3.75094	-0.41972	-0.69229	Si	3.50177	-0.45119	1.09068
		P	1.54393	-1.85975	0.78191	P	-1.45477	-2.11317	-0.35321
		P	-1.54446	-1.85929	0.78221	P	1.45472	-2.11320	-0.35322
		H	0.88350	3.94179	1.59089	H	-0.88189	4.46948	-0.49795
		H	-0.88051	3.94187	1.59198	H	0.00083	4.36668	1.02271
		H	0.00216	2.94563	2.74775	H	0.88133	4.46951	-0.49922
		H	4.66136	-0.32639	0.48849	H	-4.61760	-0.81991	0.16609
		H	4.30724	-1.46870	-1.60163	H	-3.67319	-1.26092	2.33495
		H	3.78826	0.87883	-1.42692	H	-3.62739	0.98894	1.44517
		H	1.66877	-3.16571	0.22204	H	-1.45400	-3.15491	0.62066
		H	2.44536	-1.97625	1.87327	H	-2.49690	-2.55483	-1.20602
		H	-2.44575	-1.97569	1.87369	H	2.49684	-2.55488	-1.20603
		H	-1.66991	-3.16508	0.22207	H	1.45394	-3.15495	0.62065
		H	-4.30719	-1.46736	-1.60215	H	3.67320	-1.26112	2.33487
		H	-4.66209	-0.32607	0.48838	H	4.61758	-0.81988	0.16604
		H	-3.78838	0.88012	-1.42614	H	3.62735	0.98883	1.44531
		Th	-0.00003	0.57825	-0.31735	Th	0.00001	0.61292	-0.26495
**Energy =**		-1917.0352399				- 1917.0249088			

**E**	**M**	**Opt**				**TS**			
**NCH_3**	**U**	C	0.00001	-1.20814	1.39186	C	-0.50851	-1.67496	-1.17322
		C	0.00005	3.43049	1.48670	C	1.78821	3.47802	-0.12063
		N	2.03810	-0.76218	-0.26625	N	-2.14797	-0.15156	0.35825
		N	0.00002	2.24989	0.67711	N	1.07475	2.23591	-0.21273
		N	-2.03811	-0.76216	-0.26624	N	1.62764	-1.13900	0.39277
		Si	3.62431	-0.60440	-0.91326	Si	-3.55597	0.49739	1.11030
		Si	-3.62432	-0.60440	-0.91324	Si	3.18094	-1.21802	1.14450

E	M	Opt				TS			
		P	1.51128	-1.76130	0.91587	P	-2.00975	-1.59786	-0.40596
		P	-1.51126	-1.76130	0.91587	P	0.82173	-2.34430	-0.37896
		U	-0.00001	0.57723	-0.25513	U	0.10505	0.57263	-0.24087
		H	-0.88301	4.05367	1.29838	H	2.64901	3.40028	0.55445
		H	0.88311	4.05364	1.29834	H	1.15323	4.28996	0.25542
		H	0.00006	3.18400	2.55491	H	2.17333	3.79614	-1.09703
		H	3.64478	0.60576	-1.78701	H	-3.26651	1.90083	1.52053
		H	4.66546	-0.43953	0.14643	H	-4.73222	0.50774	0.18665
		H	4.02387	-1.77631	-1.75056	H	-3.95685	-0.27015	2.32837
		H	2.47071	-1.79010	1.96141	H	-3.09700	-1.74258	-1.30443
		H	1.56062	-3.12329	0.49525	H	-2.27635	-2.66245	0.50424
		H	-1.56058	-3.12328	0.49521	H	0.51895	-3.39770	0.53189
		H	-2.47068	-1.79014	1.96140	H	1.71741	-3.00704	-1.25469
		H	-4.02388	-1.77630	-1.75053	H	3.21084	-2.22533	2.24969
		H	-4.66547	-0.43952	0.14646	H	4.24893	-1.60282	0.17225
		H	-3.64480	0.60577	-1.78699	H	3.50471	0.11670	1.71467
**Energy =**		-1986.2641936				-1986.2589884			

E	M	Opt				TS
**O**	**Ce**	C	-0.00012	1.49263	1.05435	NA
		N	-1.99204	0.30501	-0.31699	
		N	1.99156	0.30563	-0.31787	
		Si	-3.54037	-0.05461	-0.98633	
		Si	3.54039	-0.05420	-0.98596	
		P	-1.47790	1.74048	0.27686	
		P	1.47727	1.74093	0.27629	
		H	-4.65047	0.38740	-0.08650	
		H	-3.75325	0.61117	-2.30746	
		H	-3.64200	-1.52674	-1.18273	
		H	-1.46145	2.73715	-0.74338	
		H	-2.47471	2.27218	1.13399	
		H	2.47432	2.27287	1.13300	
		H	1.46012	2.73761	-0.74393	
		H	3.75481	0.61225	-2.30650	
		H	4.64980	0.38687	-0.08481	
		H	3.64161	-1.52626	-1.18306	
		O	0.00072	-2.65906	0.32845	
		Ce	0.00015	-0.89000	0.36511	
**Energy =**		-1965.0402501				

E	M	Opt				TS			
**O**	**Th**	C	0.00026	1.53737	-0.98109	C	-0.00005	1.71853	-1.13342
		N	2.08068	0.51472	0.34647	N	1.93422	0.49884	0.35129
		N	-2.08051	0.51538	0.34627	N	-1.93421	0.49874	0.35135
		Si	3.70898	0.15907	0.78650	Si	3.45443	0.21486	1.13111
		Si	-3.70864	0.15951	0.78671	Si	-3.45452	0.21483	1.13102
		P	1.51651	1.88473	-0.35652	P	1.44866	1.93539	-0.30826
		P	-1.51591	1.88520	-0.35660	P	-1.44870	1.93533	-0.30815
		Th	-0.00014	-0.78559	-0.04581	Th	0.00002	-0.79968	-0.32302
		H	4.67578	0.45712	-0.31287	H	4.59405	0.67833	0.28139
		H	4.15421	0.92040	1.99288	H	3.55424	0.93218	2.43740
		H	3.78577	-1.29501	1.11235	H	3.59718	-1.24521	1.37680

		H	1.57292	2.98510	0.54946	H	1.43844	2.93810	0.70538
		H	2.46047	2.31329	-1.32586	H	2.49905	2.40943	-1.13267
		H	-2.45969	2.31417	-1.32594	H	-2.49918	2.40940	-1.13243
		H	-1.57196	2.98558	0.54941	H	-1.43837	2.93801	0.70552
		H	-4.15379	0.92102	1.99299	H	-3.55367	0.93051	2.43826
		H	-4.67577	0.45704	-0.31249	H	-4.59386	0.68027	0.28200
		H	-3.78490	-1.29452	1.11294	H	-3.59834	-1.24545	1.37485
		O	-0.00088	-2.18814	-1.27507	O	0.00004	-2.60317	0.14928
**Energy =**		-1897.6851598				-1897.6795539			

**E**	**M**	**Opt**				**TS**			
**O**	**U**	C	-0.00009	1.65883	-1.04789	C	0.00000	1.52185	-1.13166
		N	1.95332	0.45278	0.35616	N	1.97761	0.51675	0.38384
		N	-1.95392	0.45210	0.35498	N	-1.97761	0.51675	0.38385
		Si	3.46030	0.10445	1.12956	Si	3.53921	0.21598	1.05646
		Si	-3.46004	0.10413	1.13022	Si	-3.53922	0.21598	1.05646
		P	1.46540	1.89956	-0.24421	P	1.48157	1.86574	-0.40488
		P	-1.46615	1.89906	-0.24511	P	-1.48157	1.86573	-0.40487
		H	4.60486	0.66832	0.34900	H	4.63018	0.50181	0.07559
		H	3.53046	0.68774	2.50287	H	3.79849	1.04974	2.26870
		H	3.62334	-1.36978	1.22925	H	3.61161	-1.21774	1.45310
		H	1.43453	2.88569	0.78441	H	1.48343	2.99275	0.46769
		H	2.48663	2.42252	-1.07579	H	2.48730	2.25189	-1.32682
		H	-2.48696	2.42164	-1.07743	H	-2.48731	2.25188	-1.32681
		H	-1.43641	2.88519	0.78354	H	-1.48343	2.99275	0.46770
		H	-3.52986	0.69100	2.50204	H	-3.79849	1.04971	2.26872
		H	-4.60586	0.66464	0.34908	H	-4.63017	0.50185	0.07559
		H	-3.62128	-1.37001	1.23385	H	-3.61164	-1.21775	1.45306
		U	0.00013	-0.73126	-0.35383	U	0.00000	-0.75554	-0.18103
		O	0.00018	-2.43724	0.24802	O	-0.00001	-2.50403	-0.65506
**Energy =**		-1966.9173962				-1966.9161283			

Supplementary Methods

General Experimental Procedures

All manipulations were carried out using Schlenk techniques, or an MBraun UniLab glovebox, under an atmosphere of dry nitrogen. Solvents were dried by passage through activated alumina towers and degassed before use. All solvents were stored over potassium mirrors except for ethers which were stored over activated 4 Å sieves. Deuterated solvent was distilled from potassium, degassed by three freeze-pump-thaw cycles and stored under nitrogen. $[Th\{C(PPh_2NSiMe_3)_2\}(CH_2SiMe_3)_2]$ (**3**)[1] and $[KCH_2Ph]$[2] were prepared as described previously. Triphenylmethyl amine (Ph_3CNH_2) and 2, 2-bipyridine (BIPY) were purchased from Sigma-Aldrich and was dried under dynamic vacuum for 12 hours prior to use. ^{1}H, ^{31}P, and ^{13}C NMR spectra were recorded on a Bruker 400 spectrometer operating at 400.2, 162.0, and 100.6 MHz respectively; chemical shifts are quoted in ppm and are relative to tetramethyl silane (^{1}H and ^{13}C) and external 85% H_3PO_4 (^{31}P). FTIR spectra were recorded on a Bruker Alpha spectrometer with Platinum-ATR module. UV/Vis/NIR spectrum of **5** was recorded on a Perkin Elmer Lambda 750 spectrometer. Data was collected in 1mm path length cuvette loaded in an MBraun UniLab glovebox and was run versus the toluene reference solvent.

General Computational Details

The Gaussian 09 package[3] has been used to conduct density functional theory (DFT) calculations. Two density functional approximations (DFAs) have been employed herein: the generalized gradient approximation (GGA), PBE,[4,5] and related hybrid, PBE0.[6] These DFAs are ideal for this work as PBE has recently been shown to give accurate geometries in an extensive benchmarking study of uranium organometallic systems,[7] and PBE0 is known to give improved energetics and has been previously applied by us to the study of a uranium(IV)

carbene-imido complex.[8] The f elements were treated with small core Stuttgart-Bonn quasirelativistic pseudo potentials, together with the associated segmented valence basis sets.[9-12] All other atoms were treated with Dunning's cc-pVTZ basis sets.[13] The ultrafine integration grid was employed throughout. All SCF and geometry convergence criteria were used at their default settings. Harmonic vibrational frequency calculations were conducted at all stationary point geometries. Potential spin contamination can arise in systems with unpaired electrons, and the $\langle S^2 \rangle$ expectation value provides a measure of this. Herein, the maximum deviation was found to be 0.017 for systems containing U.

Analysis of the natural localized molecular orbitals have been carried out with the NBO6 software package[14,15] interfaced with Gaussian 09. Analyses of the topology of the electron density have been performed with the AIMAll software package,[16] based on Bader's quantum theory of atoms in molecules (QTAIM).[17]

CCSD(T) calculations were performed using the Molpro 2015.1 code, with all default settings.[18] The basis sets used were as for the Gaussian DFT calculations, except that cc-pVDZ was used for H.

Supplementary References

1. Gregson, M.; Lu, E.; Mills, D. P.; Tuna, F.; McInnes, E. J. L.; Hennig, C.; Scheinost, A. C.; McMaster, J.; Lewis, W.; Blake, A. J.; Kerridge, A.; Liddle, S. T. *Nat. Commun.* **2017**, *8*, 14137.
2. Bailey, P. J.; Coxall, R. A.; Dick, C. M.; Fabre, S.; Henderson, L. C.; Herber, C.; Liddle, S. T.; Loroño-González, D.; Parkin, A.; Parsons, S. *Chem. Eur. J.* **2003** *9*, 4820.
3. Frisch, M. J.; Trucks, G. W.; Schlegel, H. B.; Scuseria, G. E.; Robb, M. A.; Cheeseman, J. R.; Scalmani, G.; Barone, V.; Mennucci, B.; Petersson, G. A.; Nakatsuji, H.; Caricato, M.; Li, X.; Hratchian, H. P.; Izmaylov, A. F.; Bloino, J.; Zheng, G.; Sonnenberg, J. L.; Hada, M.; Ehara, M.; Toyota, K.; Fukuda, R.; Hasegawa, J.; Ishida, M.; Nakajima, T.; Honda, Y.; Kitao, O.; Nakai, H.; Vreven, T.; Montgomery Jr., J. A.; Peralta, J. E.; Ogliaro, F.; Bearpark, M.; Heyd, J. J.; Brothers, E.; Kudin, K. N.; Staroverov, V. N.; Kobayashi, R.; Normand, J.; Raghavachari, K.; Rendell, A.; Burant, J. C.; Iyengar, S. S.; Tomasi, J.; Cossi, M.; Rega, N.; Millam, J. M.; Klene, M.; Knox, J. E.; Cross, J. B.; Bakken, V.; Adamo, C.; Jaramillo, J.; Gomperts, R.; Stratmann, R. E.; Yazyev, O.; Austin, A. J.; Cammi, R.; Pomelli, C.; Ochterski, J. W.; Martin, R. L.; Morokuma, K.; Zakrzewski, V. G.; Voth, G. A.; Salvador, P.; Dannenberg, J. J.; Dapprich, S.; Daniels, A. D.; Farkas, O.; Foresman, J. B.; Ortiz, J. V; Cioslowski, J.; Fox, D. J. Gaussian, Inc.: Wallingford, CT, USA 2009,.
4. Perdew, J. P.; Burke, K.; Ernzerhof, M. *Phys. Rev. Lett.* **1996**, *77*, 3865.
5. Perdew, J. P.; Burke, K.; Ernzerhof, M. *Phys. Rev. Lett.* **1997**, *78*, 1396.
6. Adamo, C.; Barone, V. *J. Chem. Phys.* **1999**, *110*, 6158.
7. Reta, D.; Ortu, F.; Randall, S.; Mills, D. P.; Chilton, N. F.; Winpenny, R. E. P.; Natrajan, L.; Edwards, B.; Kaltsoyannis, N. *J. Organomet. Chem.* **2017**, *857*, 58.
8. Lu, E.; Tuna, F.; Lewis, W.; Kaltsoyannis, N.; Liddle, S. T. *Chem. Eur. J.* **2016**, *22*,

11559.

9. Dolg, M.; Stoll, H.; Preuss, H. *J. Chem. Phys.* **1989**, *90*, 1730.

10. Küchle, W.; Dolg, M.; Stoll, H.; Preuss, H. *J. Chem. Phys.* **1994**, *100*, 7535.

11. Cao, X.; Dolg, M.; Stoll, H. *J. Chem. Phys.* **2003**, *118*, 487.

12. Cao, X.; Dolg, M. *J. Mol. Struct. (TheoChem)* **2004**, *673*, 203.

13. Dunning, T. H. J. *J. Chem. Phys.* **1989**, *90*, 1007.

14. Glendening, E. D.; Badenhoop, J. K.; Reed, A. E.; Carpenter, J E Bohmann, J. A.; Morales, C. M.; Landis, C. R.; Weinhold, F. NBO6, Theoretical Chemistry Institute, University of Wisconsin, Madison 2013.

15. Glendening, E. D.; Landis, C. R.; Weinhold, F. *J. Comput. Chem.* **2013**, *34*, 2134.

16. Keith, T. A. TK Gristmill Software: http://aim.tkgristmill.com/ **2014**.

17. Bader, R. F. W. *Atoms in Molecules: A Quantum Theory*; Oxford University Press, Oxford 1990.

18. Werner, H.-J.; Knowles, P. J.; Knizia, G.; Manby, F. R.; Schütz, M. *Wiley Interdiscip. Rev. Comput. Mol. Sci.* **2012**, *2*, 242.
